# Racial and Ethnic Disparities in Reproductive Health Care and Outcomes Among Female Veterans: A Scoping Review

**DOI:** 10.1089/heq.2024.0168

**Published:** 2025-04-10

**Authors:** Katrina S. Nietsch, Samantha L. Estevez, Nichole Goodsmith, Kristin O. Haeger, Jill Inderstrodt, Sabra S. Inslicht, Katherine A. Kosman, Qiyan Mu, Yael I. Nillni, Deirdre Quinn, Adriana Rodriguez, Lauren Siff, Krysttel C. Stryczek, Erica V. Tartaglione, Jodie G. Katon

**Affiliations:** ^1^ Icahn School of Medicine at Mount Sinai, New York City, New York, USA.; ^2^ Department of Obstetrics, Gynecology, and Reproductive Sciences, University of Pittsburgh Medical Center, Pittsburgh, Pennsylvania, USA.; ^3^ Center for the Study of Health Care Innovation, Implementation, and Policy, VA Greater Los Angeles Health Care System, Los Angeles, California, USA.; ^4^ VA Desert Pacific Mental Illness Research, Education, and Clinical Center, VA Greater Los Angeles Healthcare System, Los Angeles, California, USA.; ^5^ Department of Psychiatry and Biobehavioral Sciences, David Geffen School of Medicine at UCLA, Los Angeles, California, USA.; ^6^ US Department of Veterans Affairs, VA Office of Women’s Health, Washington, District of Columbia, USA.; ^7^ Indiana University Fairbanks School of Public Health, Indianapolis, Indiana, USA.; ^8^ San Francisco VA Health Care System, San Francisco, California, USA.; ^9^ University of California, San Francisco, San Francisco, California, USA.; ^10^ VA Boston Health Care System, Boston, Massachusetts, USA.; ^11^ Research & Simulation Division, Nursing Education, Clement J. Zablocki VA Medical Center, Milwaukee, Wisconsin, USA.; ^12^ National Center for PTSD, Women’s Health Sciences Division at VA Boston Healthcare System, Boston, Massachusetts, USA.; ^13^ Department of Psychiatry, Boston University Chobanian & Avedisian School of Medicine, Boston, Massachusetts, USA.; ^14^ Center for Health Equity Research & Promotion (CHERP), VA Pittsburgh Healthcare System, Pittsburgh, Pennsylvania, USA.; ^15^ Division of General Internal Medicine, University of Pittsburgh School of Medicine, Pittsburgh, Pennsylvania, USA.; ^16^ Virginia Commonwealth University School of Medicine, Richmond, Virginia, USA.; ^17^ Center of Innovation for the Study of Veteran-Centric and Value-Driven Care, VA Puget Sound Health Care System, Seattle, Washington, USA.; ^18^ VA Eastern Colorado Healthcare System, Aurora, Colorado, USA.

**Keywords:** reproductive health, women veterans, racial disparities, health equity, female veterans

## Abstract

**Introduction:**

Female veterans are the fastest growing group of new Veterans Health Administration (VA) users, and 40% identify as belonging to a racialized group. It is unclear if racial/ethnic disparities in reproductive health care and outcomes observed among nonveterans are present among veterans. The purpose of this scoping review was to characterize patterns of racial/ethnic disparities in reproductive health care and outcomes among female veterans.

**Methods:**

A structured PubMed search was performed to extend a prior systematic review (from 2008–2017 to 2018–2023). We included original research on reproductive health care and outcomes in female veterans that also included a measure of association to race or ethnicity. Four hundred thirty-eight articles were identified for potential inclusion. Following PRISMA guidelines, titles and abstracts were screened in duplicate, and full articles were reviewed using a standardized abstraction form. Articles were sorted into six categories by topic (contraception, infertility, pregnancy, reproductive health screenings, gynecology, and menopause) and outcomes characterized as structural (e.g., organization of care), process (e.g., access to services), or clinical/behavioral (e.g., low birthweight) measures per Donabedian’s model.

**Results:**

After title and abstract screening, 53 articles were reviewed in full. Four additional articles were excluded for a final sample of 49 articles. All articles described results from observational studies, which were almost exclusively focused on veterans using VA care (94%, *n* = 46). Topics with the greatest number of articles included pregnancy (43%, *n* = 21) and contraception (24%, *n* = 12). Racial/ethnic disparities were detected more frequently for clinical and behavioral outcome measures than for process measures.

**Conclusion:**

Consistent with literature regarding other types of VA care, racial/ethnic disparities were more prevalent for clinical and behavioral outcome measures versus process measures, highlighting that access is necessary but not sufficient for reaching health equity. Understanding the racial/ethnic health disparities and their relationships with different measures of health care quality is essential for achieving health equity for female veterans.

## Introduction

Racial and ethnic health disparities are ubiquitous in the United States, including unacceptably wide disparities in reproductive health care and outcomes across the life course for women and those assigned female at birth.^1^ For example, Black and American Indian/Alaska Native (AI/AN) birthing people have 2–3 times higher rates of pregnancy-related mortality than White birthing people. Additionally, among those with a uterus, Black and Hispanic people are less likely than non-Hispanic White people to receive any infertility treatment, have higher prevalence and symptom severity of uterine fibroids, and have higher incidence and mortality from endometrial cancer compared with White people.^2–8^ As race and ethnicity are cultural constructs, racial and ethnic disparities in reproductive health care and outcomes are a product of the historical, social, structural, and political context in which individuals live their lives in the United States.^9–12^ Concerning female reproductive health care and outcomes, racial and ethnic disparities further reflect the intersection of racism and sexism.^13–15^ Health equity, or “the state in which everyone has the opportunity to attain their full health potential and no one is disadvantaged from achieving this potential because of social position or other socially determined circumstances,” as a core component of health care quality is not new; however, in 2022, Nundy et al. proposed that health equity be added explicitly as a fifth component of the quintuple aim for health care improvement.^1,16–18^ Health equity is also a core component of the mission of the Veterans Health Administration (VA).^19,20^ Understanding where racial and ethnic disparities in health and health care exist and factors contributing to these disparities is a first necessary step toward reducing and eliminating them in VA care.

Racial and ethnic health disparities in reproductive health among female veterans are important to understand, given the rise in the number of female veterans using VA, and that this growing group of veterans is younger and more racially diverse than their male counterparts.^21^^,^^a^ Forty percent of female veterans using VA identify as belonging to a racialized group, and there is a small but growing population of female veterans who identify as Hispanic or Latina.^21,22^ Additionally, the majority of female veterans are under age 65, with approximately 40% in their childbearing years (18–45 years old), underscoring the need for VA to provide a full range of reproductive health services over the entire life course.^21,22^ Female veterans receive care through designated women’s health primary care providers, increasingly in the context of VA women’s health clinics, which may incorporate gynecology care and mental health care.^23,24^ Specialty care other than gynecology (e.g., endocrinology and orthopedics) is available in mixed-gender settings. While all VA sites offer basic reproductive health care services, and a growing number of VA sites offer both basic and specialized reproductive health care services, some services, such as obstetric care, continue to be purchased from the community due to limited demand at any one site and the resultant inability to ensure availability of necessary specialized services.^22,24,25^ Specifically, VA pays for veterans to receive obstetric care outside VA from community providers. Finally, since 2019, veterans may also more easily opt to use community care purchased by VA following passage of the (Maintaining Internal Systems and Strengthening Integrated Outside Networks) MISSION Act.^26^ Notably, the majority of veterans using VA care qualify for free care or reduced co-pays due to disability resulting from their service or low income. VA also provides benefits for veterans traveling long distances for needed care particularly veterans residing in rural areas. Thus, within VA, there are fewer financial barriers to accessing care compared with other health care systems, although veterans may still have to contend with other costs associated with time off work and costs associated with additional childcare.

Concurrent with the growth in the female veteran population and increased availability of reproductive health services in VA, there has been significant growth in the peer-reviewed literature addressing the reproductive health of female veterans.^27,28^ While a 2016 article by Carter et al. summarized the overall prevalence of racial and ethnic health disparities reported among female veterans, this review did not focus on reproductive health and precluded the recent growth in the literature.^29^ Understanding racial and ethnic disparities in reproductive health among female veterans is critical for ensuring that VA meets its goal of providing high-quality, equitable care to all veterans.

**FIG. 1.**
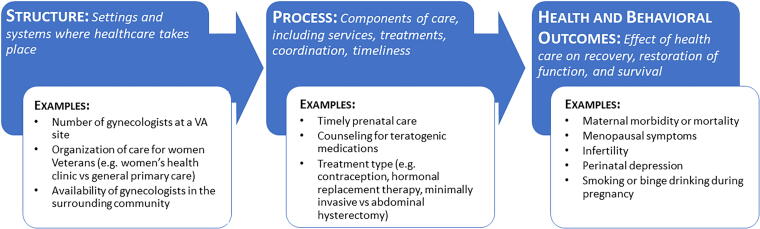
Adaptation of the Donabedian’s model of health care quality metrics for examining reproductive health care and outcomes.

The Donabedian model of health care quality measures provides a framework for examining and reporting racial and ethnic disparities across a variety of health care metrics.^30^ This model categorizes quality measures such as reflecting structures, processes, or outcomes of health care (Fig. 1). Structural measures consider the settings and systems in which health care occurs (e.g., availability of providers in the surrounding community and models of care), whereas process measures are concerned with the components of care (e.g., access, services, and treatments). Importantly, structural measures in this framework are measured at the health care system level or at the level of a geographic catchment area. Clinical and behavioral outcome measures reflect the effect of health care on recovery, restoration of function, and survival (e.g., maternal mortality and perinatal depression).^30^ Using this framework for health care quality to assess racial and ethnic disparities in VA can help us understand the causes of these disparities and inform strategies for achieving health equity. For example, if Black pregnant veterans are more likely to live in areas with fewer obstetrics providers (structure), they might be less likely to receive timely prenatal care (process). Additionally, bias might lead their providers to be less attentive and less likely to ensure they receive all necessary prenatal care and testing (process). Independently and cumulatively, these factors could result in an increased risk of maternal morbidity and mortality among pregnant and birthing Black veterans (clinical outcome). Such findings would suggest a need for programs and policies to improve pre-pregnancy health and increase access to obstetric providers as well as ensure that the content of this care and patient experience meets specific quality measures with the goal of reducing racial and ethnic disparities in maternal morbidity and mortality. Therefore, the purpose of this scoping review was to examine the evidence concerning racial and ethnic disparities in reproductive health among female veterans, applying Donabedian’s framework to characterize patterns in these disparities and their implications for policy and practice and identifying critical topical and methodologic gaps in the existing literature.^30^

## Methods

We used a search strategy adapted from an earlier systematic review of the literature on the reproductive health of female veterans led by Dr. Katon.^27,28^ We included all articles from the prior systematic review that included articles from 2008 to 2017.^28^ Based on our prior knowledge of the literature, we initially searched the literature to identify any articles addressing the reproductive health and health care of women veterans. We then reviewed titles and abstracts to identify those that potentially included findings regarding racial or ethnic disparities. We did not search the literature before 2008, as the earlier evidence report and systematic review indicated only three articles addressing reproductive health of women veterans were published between 2008 and 2011 and a review of the literature prior to 2008 found even fewer articles on this topic.^27,31^ We ran a structured search in PubMed using identical terms as the prior systematic review to update the article list to include those published between 2018 and 2023 (search run August 14, 2023). We also ran a second modified version of the first search in PubMed, adding the term “female.” Finally, we reviewed editorials, case reports, review articles, and reference lists to identify any additional articles. In this way, we could capture articles that included findings on racial or ethnic disparities even when this was not the primary focus of the article. Our final sample included all original research articles, published between 2008 and 2023, addressing reproductive health care or outcomes of female veterans that also included outcome frequencies by race or ethnicity or measures of association for race or ethnicity with the outcome of interest. Following Preferred Reporting Items for Systematic reviews and Meta-Analyses (PRISMA) guidelines, once the complete list of articles was identified, two co-authors (JK and KN) reviewed titles and abstracts to identify a subset of articles for full review.^32^ When there was disagreement regarding inclusion/exclusion, a third author was brought in to adjudicate (LS). Articles were reviewed in full using a standardized abstraction form (see Supplementary Data) with 59% undergoing review from two or more co-authors to ensure consistency. While details such as assessment of study quality and findings were beyond the scope of this review, we collected these data as part of the abstraction process to enable future systematic reviews and meta-analyses if enough studies on a given topic were identified. We used Covidence software to track search results and exclusions.^33^

For synthesis, articles were sorted into six categories based on topic (contraception, infertility, pregnancy, reproductive health screenings, gynecology, and menopause), and outcomes were defined as structural, process, or clinical or behavioral outcome measures.^30^ Given the growing call to acknowledge the role of racism in determining racial and ethnic health disparities, we also carefully reviewed article texts to determine if the term racism was used in any part of the article and whether race or ethnicity was the primary independent variable of interest.^34^

## Results

**FIG. 2.**
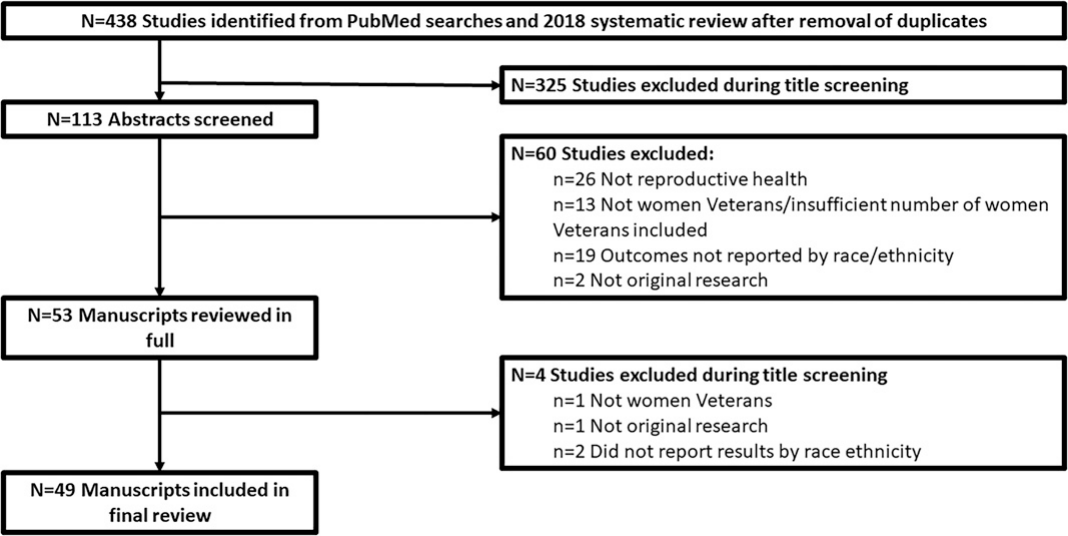
PRISMA diagram of inclusions and exclusions.

After combining the article list from the prior systematic review with the results from the two searches and removing duplicates, 438 articles remained. Our final sample included 49 articles (Fig. 2). All studies included were observational, including one qualitative study, and nearly all exclusively focused on veterans using VA (94%, *n* = 46; Table 1), with half relying solely on VA administrative data (51%, *n* = 25). Among the three studies not focused on veterans using VA, two relied on a sampling frame generated by combining VA and Department of Defense data^35,36^ and one used data from a Centers for Disease Control and Prevention (CDC) national US population-based survey that included veterans and nonveterans.^37^ Among those focused on veterans using VA, the period of use varied significantly between studies ranging from 2001 to 2020. The topics with the greatest number of articles included pregnancy (43%, *n* = 21) and contraception (24%, *n* = 12). Only two articles included at least one structural measure (8%), and the remainder were split nearly evenly between process measures (53%, *n* = 26) and clinical and behavioral outcomes (53%, *n* = 26). Five articles (10%) included process and clinical or behavioral outcome measures. Only 35% of articles included race and ethnicity as the primary independent variable (*n* = 17) and 27% (*n* = 13) named racism as a cause for racial disparities. Additionally, few if any articles included measures of racism at the interpersonal, institutional, or systemic level. These factors limited discussion of potential mechanisms underlying racial disparities, particularly how racism might operate at multiple levels (e.g., individual, institutional, and systemic) to produce health disparities among female veterans. Racial or ethnic disparities were reported more frequently for clinical and behavioral outcome measures (96%) versus process measures (65%). Below we summarize results by topic and type of measure. Notably, there was tremendous heterogeneity in terminology and categorization of racial and ethnic groups throughout the articles reviewed; when describing results in published articles, we have tried to stay consistent with terms used in the original articles; this includes terminology that is no longer widely accepted. However, we use the more widely accepted language when discussing and synthesizing findings.

**Table 1.** Characteristics of Included Articles (*n* = 49)

	*n*	%
Type of study		
Cross-sectional	16	33
Cohort/longitudinal	32	65
Qualitative	1	2
Population		
Veterans using VA health care	46	94
Data source		
VA administrative data	25	51
Survey data	23	47
Semistructured interviews	1	2
Topic area		
Pregnancy	21	43
Contraception	12	24
Gynecology	7	14
Reproductive health screenings	3	6
Menopause	3	6
Infertility	3	6
Type of outcome measure^a^		
Structure	2	7
Process	26	53
Clinical or behavioral	26	53
Race and ethnicity		
Race or ethnicity primary independent variable	17	35
Racism mentioned in article	13	27

^a^ Includes five articles with both process and clinical or behavioral outcomes.

VA, Veterans Health Administration.

### Pregnancy

Table 2 details the articles addressing pregnancy by measure type (*n* = 21). Two articles are listed twice as they included both process and clinical or behavioral measures.^45,46^ Forty-three percent (*n* = 9) of the articles used data from the Center for Maternal and Infant Outcomes Research in Translation^39^ study, a prospective cohort study that enrolled female veterans early in pregnancy.

**Table 2.** Articles that Addressed Measures of Health Care Quality for Pregnancy, by Measure Type and Year

Measure type	Primary author (year)	Study design	Data source	Population	*N*	Outcome(s)	Findings
Process	Kroll-Desrosiers (2016)^38^	Cross-sectional	VA administrative data	Women veteran VHA users ages 18–45 with a VA paid delivery 2001–2010	*n* = 2331 (White *n* = 1274, Black *n* = 461, other *n* = 479, unknown *n* = 115; Hispanic/Latinx ethnicity *n* = 298, non-Hispanic/Latinx ethnicity *n* = 1916, unknown ethnicity *n* = 115)	At least one filled prescription for opioids in the VHA prescription data in the 280 days before delivery	No difference in receipt of opioid prescription during pregnancy by race or ethnicity
Process	Mattocks (2019)^39,a^	Cross-sectional	Survey data	Pregnant veterans using the VA Maternity Care Coordination program, enrolled from 15 VA facilities nationwide, participating in telephone surveys at 20 weeks of pregnancy	*n* = 519 (non-Hispanic/Latinx White *n* = 319, non-Hispanic/Latinx Black *n* = 123, other *n* = 85 [race not mutually exclusive]; Hispanic/Latinx ethnicity *n* = 87)	Perceived timely access to prenatal care based on response to survey question: “Did you receive prenatal care as early as you wanted it?”	Non-Hispanic/Latinx White veterans were more likely to report perceived timely access to prenatal care than racial/ethnic minority veterans (66% versus 52%; *p* = 0.0038)
Process	Grekin (2020)^40,a^	Cohort/longitudinal	Survey data	Women veterans who were VA users and part of a pregnancy cohort	*n* = 510 (White *n* = 321, non-White *n* = 189)	Intention to use VA care during pregnancy, actual use of VA care during pregnancy	No association between race and intent to use VA care (adjusted odds ratio (aOR) 0.81; 95% confidence Interval (CI: 0.34 − 1.19) or actual use of VA care during pregnancy (aOR 0.74; 95% CI: 0.50 − 1.09)
Process	Mattocks (2021)^41,a^	Cross-sectional	Survey data	Veterans using the VA Maternity Care Coordination program, enrolled from 15 VA facilities nationwide who completed a postpartum interview	*n* = 659 (White *n* = 398, other *n* = 261; Hispanic/Latinx ethnicity *n* = 123)	Cesarean section	Compared with White woman veterans, woman veterans of other races were more likely to have a cesarean versus a vaginal birth (aOR 1.67; 95% CI: 1.13 − 2.48). No differences by Hispanic/Latinx ethnicity
Process	Shankar (2021)^42^	Cohort/longitudinal	VA administrative data	Veterans with VA-paid obstetric inpatient claims for fiscal years 2005–2014	*n* = 18,414 (non-Hispanic/Latinx White *n* = 10,641, non-Hispanic/Latinx Black *n* = 4180, Asian *n* = 312, Native Hawaiian/other Pacific Islander *n* = 174, American Indian/Alaska Native *n* = 172, Hispanic/Latinx *n* = 1915, unknown *n* = 1020)	Any VA primary care visit within 1 year following childbirth	Compared with non-Hispanic/Latinx White veterans, non-Hispanic/Latinx Black (aOR 0.88; 95% CI: 0.82 − 0.94) and Asian (aOR 0.66; 95% CI: 0.53 − 0.83) veterans were less likely to have a postpartum VA primary care visit
Process	Copeland (2022)^43,a^	Cohort/longitudinal	VA administrative data and survey data	Women veterans who were VA users and part of a pregnancy cohort with VA pharmacy data available before conception, during pregnancy, and postpartum	*n* = 501 (Native American *n* = 8, Black *n* = 159, Native Hawaiian or Pacific Islander *n* = 11, White *n* = 314); ethnicity collected separately (Hispanic/Latinx *n* = 56)	Prescription for “potentially risky” medications immediately before pregnancy, during pregnancy, or postpartum; discontinuation of antidepressants (SSRI/SNRIs) during pregnancy	Race was not associated with receipt of prescription of potentially risky medications during pregnancy. Black race was associated with an increased risk of discontinuation of antidepressants during pregnancy (adjusted relative risk ratio (RRR) = 1.41; 95% CI: 1.0 − 1.85)
Process	Sheahan (2022)^44,a^	Cross-sectional	Survey data	Veterans who were pregnant between January 2016 and September 2021 were identified by site leads and invited to participate in a telephone survey during pregnancy	*n* = 851 (White *n* = 479, Black *n* = 198, other *n* = 174; Hispanic/Latinx ethnicity *n* = 168)	Sufficiency of health information during pregnancy based on a composite score calculated by summing responses to nine health topics	No detected association between race or ethnicity and sufficiency of health information received during pregnancy
Process	Katon (2023)^45,b^	Cross-sectional	Survey data	Veterans with a VA paid live birth between June 2018 and December 2019	Weighted totals: *n* = 3439 (Black *n* = 1027, White *n* = 2412). 12.9% identified as Hispanic/Latinx with 5.0% data missing.	Process outcomes: (1) timely initiation of prenatal care (initiation before 13 weeks gestation, yes/no), (2) perceived access to timely prenatal care (whether or not a participant got prenatal care as early in their pregnancy as they would have liked, yes/no), (3) attendance at a postpartum check-up (yes/no), and (4) cesarean section	No racial disparities in access or use of care were detected, including timely prenatal care, needed mental health care, or postpartum visit
Process	Kinney (2023)^46,a,b^	Cross-sectional	Survey data	English-speaking veterans, birth sex female, 18 years of age or older, with a confirmed pregnancy and enrolled in VA care at one of the 15 COMFORT study sites throughout the United States	*n* = 829 infants born to *n* = 811 veterans (non-Hispanic White *n* = 421, Minority race *n* = 390)	Newborn insurance coverage	Maternal minority race or ethnicity not associated with infant insurance coverage (results not provided)
Clinical or behavioral	Shaw (2014)^47^	Cohort/longitudinal	VA administrative data	Veterans with VA paid deliveries in fiscal years 2000–2012	*n* = 16,334 deliveries among 14,047 veterans (White *n* = 10,262, Black *n* = 3673, Asian *n* = 256, Native Hawaiian or other Pacific Islander *n* = 233, American Indian or Alaska Native *n* = 130, unknown/missing *n* = 1780)	Spontaneous preterm birth before 37 weeks gestation by ICD-9	Compared with White veterans, Black veterans were more likely to have a spontaneous preterm birth (aOR 1.49; 95% CI: 1.29 − 1.71)
Clinical or behavioral	Kroll-Desrosiers (2019)^48,a^	Cross-sectional	Survey data	Pregnant women veterans using VA health care	*n* = 501 (White *n* = 310, Black *n* = 116, other *n* = 83; Hispanic/Latinx *n* = 85)	Depression symptoms as indicated by a EPDS score of >10	No association between non-White race and depression symptoms (aOR 0.74; 95% CI: 0.44 − 125)
Clinical or behavioral	Combellick (2020)^49^	Cross-sectional	VA administrative data and chart review	Women veterans deployed in service to OEF/OIF/OND with a VA paid birth 2014–2016 with severe maternal morbidity	*n* = 9895 (non-Hispanic/Latinx White *n* = 5088, non-Hispanic/Latinx Black *n* = 2678, Hispanic/Latinx *n* = 1316, other *n* = 809, unknown *n* = 4)	Severe maternal morbidity defined by diagnostic codes for 18 conditions used by CDC	Compared with those without severe maternal morbidity, a higher percentage of those with severe maternal morbidity were non-Hispanic/Latinx Black (27% versus 44%)
Clinical or behavioral	Kedem (2020)^50,a^	Cross-sectional	Survey data	Postpartum women veterans recruited during pregnancy from 15 VA facilities	*n* = 420 (White *n* = 269, Black *n* = 86, Other *n* = 65; Hispanic/Latinx ethnicity *n* = 61)	Breastfeeding for four or more weeks postpartum	Compared with White veterans, Black veterans were less likely to breastfeed four or more weeks postpartum (aOR 0.48; 95% CI: 0.24 − 0.99). No differences by Hispanic/Latinx ethnicity
Clinical or behavioral	Nilni (2020)^51^	Cohort/longitudinal	Survey data	Women veterans who were part of a nationwide prospective cohort study who became pregnant in the first 3 years after separating from US military service	*n* = 318 (non-Hispanic/Latinx White *n* = 198, non-White *n* = 120)	Adverse birth outcomes, postpartum depression/anxiety, perceived difficult pregnancy	Non-White veterans had a decreased risk of experiencing an adverse pregnancy outcome (aOR 0.58; 95% CI: 0.34 − 0.97) and increased likelihood of perinatal PTSD symptoms, no differences detected for self-reported postpartum depression/anxiety or perception of pregnancy difficulty
Clinical or behavioral	Albright (2021)^37^	Cross-sectional	CDC BRFSS (2016, 2017, 2018)	Pregnant respondents	*n* = 6101 (Veteran White *n* = 68, racial/ethnic minority (reported as White non-Hispanic, Black non-Hispanic, American Indian or Alaskan Native non-Hispanic, Asian non-Hispanic, Native Hawaiian or other Pacific Islander non-Hispanic, multiracial non-Hispanic, Hispanic, or other non-Hispanic and further categorized as White and racial/ethnic minority for the analysis) *n* = 77; non-Veteran White *n* = 3763, racial/ethnic minority *n* = 2193)	Binge drinking during pregnancy (self-report past 30 days)	Binge drinking during pregnancy prevalence 3.6%; prevalence highest among racial/ethnic minority veterans (17.4%) versus White veterans (5.34%) versus racial/ethnic minority non-veterans (4.1%) versus White nonveterans (3%)
Clinical or behavioral	Colman (2021)^52^	Cross-sectional	VA administrative data for veterans included in the Women Veterans Cohort Study (WVCS)	Women veterans from WVCS with a VA paid birth and smoking health factor data within 280 days of birth	*n* = 6190 (did not provide racial distribution, includes Black non-Hispanic/Latinx, Hispanic/Latinx, other/unknown, White non-Hispanic/Latinx)	Prenatal smoking (positive smoking health factor in the 280 days before birth)	White (ref: non-White) race was associated with higher likelihood of prenatal smoking (aOR 2.30 (CI 1.90 − 2.70), *p* < 0.05).
Clinical or behavioral	Kroll-Desrosiers (2021)^53,a^	Cross-sectional	Survey data	Pregnant women veterans using VA health care who reported smoking at the beginning of pregnancy	*n* = 133 (White *n* = 91, Black *n* = 26, other *n* = 20; Hispanic/Latinx ethnicity *n* = 19)	Smoking during pregnancy: Persistent smoker versus quit	No association between race or ethnicity and smoking during pregnancy
Clinical or behavioral	Lumsden (2022)^54,a^	Cohort/longitudinal	Survey data	Pregnant women veterans using VA and enrolled in COMFORT between Jan. 28, 2016-Jan. 29, 2020	*n* = 706 (White *n* = 422, Black *n* = 155, non-Black (Asian, Native Hawaiian or other Pacific Islander, American Indian or Alaska Native, or other) *n* = 129, all not mutually exclusive; Hispanic/Latinx ethnicity *n* = 136, non-Hispanic/Latinx ethnicity *n* = 567)	Pregnancy-related cardiovascular condition (preterm birth, pre-eclampsia/eclampsia, and/or pregnancy-related hypertension, gestational diabetes)	Frequency of pre-pregnancy risk factors for pregnancy-related CVD differed by race
Clinical or behavioral	Katon (2023)^45,b^	Cross-sectional	Survey data	Veterans with a VA paid live birth between June 2018 and December 2019	Weighted totals: *n* = 3439 (Black *n* = 1027, White *n* = 2412). 12.9% identified as Hispanic/Latinx with 5.0% data missing.	Clinical outcomes included (1) postpartum rehospitalization, (2) low birthweight, (3) preterm birth, (4) admission to a neonatal intensive care unit (NICU) after birth, and (5) breastfeeding	Black veterans were more likely than White veterans to have a postpartum rehospitalization (RR 1.67; 95% CI: 1.04 − 2.68) and a low-birthweight infant (RR 1.67; 95% CI: 1.20 − 2.33)
Clinical or behavioral	Kinney (2023)^46,a,b^	Cross-sectional	Survey data	English-speaking veterans, birth sex female, 18 years of age or older, with a confirmed pregnancy and enrolled in VHA care at one of the 15 COMFORT study sites throughout the United States	*n* = 829 infants born to *n* = 811 veterans (non-Hispanic White *n* = 421, Minority race *n* = 390)	Preterm birth, low birth weight	Compared with non-Hispanic White VHA users, those who identified as a racial or ethnic minority were more likely to give birth to an infant with low birthweight (<5.8 pounds; aOR 2.3; 95% CI: 1.3 − 4.0, *p* < 0.003), but no more or less likely to have a preterm birth (aOR 1.1; 95% CI: 0.7 −1.8, *p* < 0.73)
Clinical or behavioral	Panelli (2023)^55^	Cohort/longitudinal	VA administrative data	Women veterans using VA with singleton livebirth with a diagnosis of PTSD within 12 months before childbirth	*n* = 2293 veterans and *n* = 3242 births (non-Hispanic/Latinx White *n* = 2091, non-Hispanic/Latinx Black *n* = 685, Hispanic/Latinx *n* = 357, other *n* = 109)	Spontaneous preterm birth before 37 weeks gestation by ICD-9	Race/ethnicity not associated with spontaneous preterm birth. Non-Hispanic/Latinx Black (aOR 1.23; 95% CI: 0.90–1.69), Hispanic (aOR 1.07; 95% CI: 0.69–1.66), other (aOR 1.00; 95% CI: 0.47–2.12)
Clinical or behavioral	Pratt (2023)^36^	Cohort/longitudinal	Survey data	Women veterans 20–45 years old separated from service within the last 10 years with a functional uterus at some point in time, have ever been pregnant, and answered at least one of the postpartum diagnoses’ questions (PPD, PPA, PPPTSD)	*n* = 1039 (Asian or Pacific Islander *n* = 15, non-Hispanic/Latinx Black *n* = 130, Hispanic/Latinx *n* = 51, Native American/Alaska Native *n* = 6, White *n* = 559, Multiple *n* = 273)	Self-reported diagnosis of perinatal depression, anxiety, or PTSD	Race/ethnicity not associated with diagnosis of perinatal depression or anxiety. Compared with non-Hispanic White veterans, non-Hispanic/Latinx Black veterans (aOR 2.0; 95% CI: 1.1–3.7), and multi-racial veterans (aOR 1.9; 95% CI: 1.2–2.9) were at an increased risk of diagnosis of perinatal PTSD
Clinical or behavioral	Shapiro (2023)^56,a^	Cross-sectional	Survey data	Veterans who were pregnant between January 2016 and September 2021 were identified by site leads and invited to participate in a telephone survey during pregnancy	*n* = 1324 (White *n* = 759, Black *n* = 360, other reported race *n* = 253; Hispanic/Latinx ethnicity *n* = 260, non-Hispanic/Latinx ethnicity *n* = 1054)	Depression symptoms as indicated by a EPDS score of >10	Compared with White veterans, Black veterans were more likely to experience prenatal depression symptoms (aOR 1.90; 95% CI: 1.42–2.54)

^a^ Used data from the Center for Maternal and Infant Outcomes Research in Translation (COMFORT) study.

^b^ Study included multiple types of measures.

aOR, Adjusted odds ratio ; COMFORT, Center for Maternal and Infant Outcomes Research in Translation study; EPDS, Edinburgh Postnatal Depression Scale; 95% CI, 95% Confidence Interval; PPA, Postpartum anxiety; PPD, Postpartum depression; PPPTSD, Postpartum posttraumatic stress disorder.

#### Pregnancy | process measures (n = 13)

Process measures examined included measures of patient-reported access, patient-reported and electronic health record-determined health care use, and measures of content and type of care. Patient-reported access measures included timely access to prenatal care (initiation before the end of the first trimester),^45^ perceived timely access to prenatal care (got prenatal care as early as desired),^39,45^ access to mental health care during pregnancy,^45^ and infant insurance coverage.^46^ Measures of patient-reported health care use included intent to use VA care during pregnancy^40^ and attendance at a 6–8-week postpartum visit,^45^ whereas electronic health records were used to determine whether a veteran had a VA primary care visit in the 12 months postpartum.^42^ In terms of content and type of treatment, measures included sufficiency of health information received during pregnancy,^44^ receipt of an opioid prescription^38^ or one for other risk medications during pregnancy,^43^ and having a cesarean section versus a vaginal birth.^41,45^ Studies relying on patient-reported measures varied in terms of sampling frames (convenience versus population-based), study designs (prospective cohort versus cross-sectional survey), and population size.^39,45^ Not incorporated into any included study were measures of patient-reported experience, particularly measures of obstetric racism or mistreatment during birth, which in the general population are disproportionately reported by Black birthing people.^57–59^

#### Pregnancy | behavioral or clinical measures (n = 14)

A wide variety of maternal behavioral health measures were used, including perinatal depression symptoms;^48,56^ diagnosed perinatal depression, anxiety, or post-traumatic stress disorder;^36,51^ prenatal smoking;^52,53^ binge drinking during pregnancy;^37^ and breastfeeding.^45,50^ Maternal clinical outcomes included preterm birth,^45–47,55^ nontraditional cardiovascular risk factors during pregnancy,^54^ and severe maternal morbidity.^49^ The only clinical infant measure reported was maternal report of low birth weight.^45,46^ Studies regarding racial disparities in behavioral and clinical measures varied widely in terms of how measures were operationalized, the population size, sampling frames, and operationalization of racial categories.

### Contraception

Table 3 details the articles addressing contraception by measure type (*n* = 12). Fifty percent (*n* = 6) of the articles relied on data from the Examining Contraceptive Use and Unmet Need, a national cross-sectional survey of a random sample of women veterans of childbearing age (18–45 years old).^72^ Notably, the majority of these articles were conceived of and published prior to or in the midst of a paradigm shift in contraceptive care to an approach emphasizing patient-centered communication and an increasing understanding of the limitations of assessing pregnancy intention and harms of focusing solely on provision and uptake of long-acting reversible contraception.^73–76^

**Table 3.** Articles that Addressed Measures of Health Care Quality for Contraception, by Measure Type and Year

Measure type	Primary author (year)	Study design	Data source	Population	*N*	Outcome(s)	Findings
Process	Borrero (2012)^60^	Cross-sectional	VA administrative data	Women veterans 18–45 years old with ≥1 VA primary care visit in fiscal year (FY) 2008	*n* = 103,950 (Hispanic *n* = 6549; non-Hispanic White *n* = 41,892; non-Hispanic African American *n* = 25,468; non-Hispanic other *n* = 3015; missing race/ethnicity *n* = 27,026)	Any contraceptive method documented; type of contraceptive method (most effective, moderately effective, least effective)	Compared with non-Hispanic/Latinx White women, Hispanic (OR: 0.82; 95% CI: 0.76–0.88) and non-Hispanic/Latinx Black women (OR: 0.85; 95% CI: 0.81–0.89) were less likely to have a documented contraceptive method. Compared with non-Hispanic/Latinx White women, non-Hispanic/Latinx Black women were more likely to have an effective method (OR: 1.10; 95% CI: 1.01 −1.20)
Process	Schwarz (2013)^61^	Cross-sectional	Survey data	Female veterans of Operation Iraqi Freedom and/or Operation Enduring Freedom enrolled for VA care in the New England or Indiana region who completed a survey between 2008 and 2010	*n* = 286 (non-Hispanic/Latinx White *n* = 239, non-Hispanic/Latinx Black *n* = 17, Hispanic/Latinx *n* = 25, Asian or Pacific Islander *n* = 5)	Received counseling about risk of medication-induced birth defects (Y/N)	No difference in receipt of counseling regarding teratogenic medications by race (*p* = 0.94)
Process	Callegari (2017)^62,a^	Cross-sectional	Survey data	Women veterans 18–45 years old with ≥1 VA primary care visit in the past year	*n* = 2302 (non-Hispanic/Latinx White *n* = 1188, non-Hispanic/Latinx Black *n* = 665, Hispanic/Latinx *n* = 285, other *n* = 164)	Contraception preferences, beliefs, and self-efficacy measured by responses to questions adapted from the National Longitudinal Study of Adolescent Health with Likert scale response options	Compared with non-Hispanic/Latinx Whites, non-Hispanic/Latinx Blacks, and Hispanic/Latinx veterans were less likely to consider contraceptive effectiveness extremely important (aOR 0.55; 95% CI: 0.40–0.74) and more likely to rate does not contain any hormones (aOR 1.94; 95% CI: 1.56–2.41; aOR 1.72; 95% CI: 1.29–2.28) and prevents sexually transmitted infections (aOR 1.99; 95% CI: 1.57–2.51; aOR 1.63; 95% CI: 1.21–2.19) as extremely important
Process	Gawron (2022)^63^	Cohort/longitudinal	VA administrative data	Emergency contraceptive (EC) users	*n* = 4280 EC prescriptions for *n* = 3120 veterans (analysis at prescription level); Race: Black *n* = 1605, White *n* = 2022, other *n* = 297, missing/unknown *n* = 356; Ethnicity: Hispanic/Latinx *n* = 662, non-Hispanic/Latinx *n* = 2298, missing/unknown *n* = 220	Type of EC prescription (levonorgestrel or ulipristal acetate)	Being White (2.10; 95% CI: 1.45–3.03) and non-Hispanic/Latinx (1.72; 95% CI: 1.15–2.57) were associated with increased likelihood of prescription of ulipristal acetate versus levonorgestrel
Clinical or behavioral	Borerro (2013)^64^	Cohort/longitudinal	VA administrative data	Women VA users 18–45 years old with hormonal contraceptive coverage in the first week of fiscal year 2008.	*n* = 6946 (non-Hispanic/Latinx White *n* = 3265, non-Hispanic/Latinx Black *n* = 1528, Hispanic/Latinx *n* = 347, other *n* = 215, unknown or missing race/ethnicity *n* = 1528)	Any gaps in coverage ≥7 days; time to first gap	Compared with non-Hispanic/Latinx White women, Hispanic/Latinx women were more likely to experience gaps in contraceptive coverage (HR 1.18; 95% CI: 1.03–1.34, *p* = 0.02)
Clinical or behavioral	Kazerooni (2014)^65^	Cohort/longitudinal	VA administrative data	Female veterans VA users, 18–44 years old, in VISN 22, who newly started hormonal contraception (pill, patch, or ring only) between October 2008 and September 2012	*n* = 3622 (White *n* = 2083, Black or African American *n* = 581, Asian *n* = 124, other/unknown *n* = 621; Hispanic/Latinx ethnicity *n* = 823, non-Hispanic/Latinx ethnicity *n* = 2799)	Adherence based on medication possession ratio (MPR) defined as the ratio of days supply dispensed divided by days in a given time interval (365 days used as denominator in this study)	Being Black (aOR 0.73; 95% CI: 0.44–1.22) or Hispanic (aOR 0.73; 95% CI: 0.46–1.14) was associated with lower MPR
Clinical or behavioral	Kazerooni (2015)^66^	Cohort/longitudinal	VA administrative data	Female veterans VA users, 18–44 years old, in Southern California and Nevada, who newly started hormonal contraception (pill, patch, or ring only) between October 2008 and September 2012	*n* = 2166 (White *n* = 1247, Black *n* = 361, Asian *n* = 121, Native American *n* = 64, unknown *n* = 373; Hispanic/Latinx ethnicity *n* = 518, non-Hispanic/Latinx ethnicity *n* = 1648)	Pregnancy or any event related to a pregnant state (e.g., positive pregnancy test, miscarriage, in a state of pregnancy, child birth, or abortion) occurring within 1 year of index date by ICD-9	No association or race or ethnicity with pregnancy after start of hormonal contraception
Clinical or behavioral	Rosenfeld (2017)^67,a^	Cross-sectional	Survey data	Women veterans 18–44 years old who used VA primary care in the prior 12 months	*n* = 2302 (non-Hispanic/Latinx White *n* = 1188, non-Hispanic/Latinx Black *n* = 665, Hispanic/Latinx *n* = 285, other *n* = 164)	Knowledge of contraceptive options measured by percent of questions answered correctly	Non-Hispanic/Latinx Black and Hispanic/Latinx women had lower overall contraceptive knowledge scores than non-Hispanic/Latinx White women (51% and 53% versus 58%, overall *p* < 0.001)
Clinical or behavioral	Rosenfeld (2018)^68,a^	Cross-sectional	Survey data	Women veterans 18–44 years old who used VA for primary care in the previous 12 month and did not have prior sterilization/hysterectomy	*n* = 1241 (non-Hispanic/Latinx White *n* = 631, non-Hispanic/Latinx Black *n* = 349, Hispanic/Latinx *n* = 166, other *n* = 95)	Reproductive coercion: Positive response to any of male partner reproductive coercion items	Compared with non-Hispanic/Latinx White veterans, non-Hispanic/Latinx Black veterans (aOR 2.69; 95% CI: 1.69–4.27) and those of other racial groups (aOR 2.79; 95% CI: 1.54–5.71) were more likely to report experiencing reproductive coercion. No difference between non-Hispanic/Latinx White and Hispanic/Latinx veterans was detected (aOR 0.84; 95% CI: 0.41–1.69)
Clinical or behavioral	Koenig (2019)^69,a^	Cross-sectional	Survey data	Women veterans ages 18–44 receiving primary care in VA	*n* = 987 (non-Hispanic/Latinx White *n* = 508, non-Hispanic/Latinx Black *n* = 274, Hispanic/Latinx *n* = 132, other *n* = 73)	Self-reported use of either an IUD or implant, at the time of last vaginal intercourse	Compared with non-Hispanic/Latinx White veterans, Hispanic/Latinx veterans were less likely to use an IUD or implant at last intercourse (aOR 0.58; 95% CI: 0.3–0.93)
Clinical or behavioral	Judge-Golden (2020)^70,a^	Cross-sectional	Survey data	Women veteran VA users at high risk of “unintended pregnancy”	*n* = 979 (non-Hispanic/Latinx White *n* = 538, non-White *n* = 441)	Match between self-reported current contraceptive method and ideal method	Non-White race/ethnicity (aOR 0.68; 95% CI: 0.52–0.89) was negatively associated with ideal–current match
Clinical or behavioral	Quinn (2020)^71,a^	Cross-sectional	Survey data	Women veteran VA users, 18–44 years old who accessed VA primary care in the past 12 months (ECUUN parent study)	*n* = 2302 (non-Hispanic/Latinx White *n* = 1188, non-Hispanic/Latinx Black *n* = 665, Hispanic/Latinx *n* = 285, other *n* = 164)	Self-reported (1) history of unintended pregnancy, (2) use of contraception at last intercourse, and (3) use of prescription method at last intercourse	Compared with non-Hispanic/Latinx White veterans, Hispanic/Latinx (aOR 1.58; 95% CI: 1.13–2.19), non-Hispanic/Latinx Black (aOR 1.81; 95% CI: 1.39–2.36), and non-Hispanic/Latinx other veterans (aOR 1.58; 95% CI: 1.04–2.34) were more like to have a history of unintended pregnancy. Compared with non-Hispanic/Latinx White veterans, Hispanic/Latinx veterans were less likely to have used prescription contraception at last intercourse (aOR 0.52; 95% CI: 0.35–0.77)

^a^ Used data from the Examining Contraceptive Use and Unmet Need (ECUUN) study.

#### Contraception | process measures (n = 4)

Process measures related to contraception included self-reported preconception counseling regarding teratogenic medications,^61^ receipt of any contraception^60^ and type of emergency contraception as documented in the electronic health record,^63^ and self-reported contraceptive preferences.^62^ While not traditional process measures, findings regarding differences in contraceptive preferences by race provide important context for understanding findings regarding racial disparities for behavioral and clinical.

##### Contraception: behavioral or clinical measures (n = 8)

In terms of behavioral and clinical measures, there was an emphasis on contraceptive adherence, unintended pregnancy, and use of long-acting reversible contraception. Three separate articles used various electronic health record-based operationalizations of adherence,^64–66^ while one study examined self-report of lifetime history of unintended pregnancy,^71^ and another looked at the use of an intrauterine device (IUD) or contraceptive implant at last intercourse.^69^ Two separate studies focused on differences by race and ethnicity in contraceptive knowledge^67^ and concordance between self-reported ideal contraceptive method and current method.^70^ Finally, one study examined self-reported experiences of reproductive coercion.^68^

### Gynecology

Of the seven articles on gynecology, two addressed structural measures,^77,78^ and five addressed process measures (Table 4).^79–83^ None addressed behavioral or clinical measures. The majority of measures were derived from a combination of electronic health record data and VA organizational data.^77,78,80,81,83^ Exceptions included one article that relied on survey data^79^ and a qualitative article based on semistructured interviews with Black veterans with symptomatic uterine fibroids.^82^

**Table 4.** Articles that Addressed Measures of Health Care Quality for Gynecology, by Measure Type and Year

Measure type	Primary author (year)	Study design	Data source	Population	*N*	Outcome(s)	Findings
Structural	Katon (2019)^78^	Cross-sectional	VA administrative data and WATCH	Women veterans with a hysterectomy at VA between FY12 and FY15	*n* = 1255 (Black *n* = 766, White *n* = 489)	Determinants of minimally invasive versus abdominal hysterectomy among Black versus White veterans	Black veterans were more likely than White veterans to undergo hysterectomy for uterine fibroids at VA medical facilities with characteristics likely to promote the use of minimally invasive surgery
Structural	Friedman (2022)^77^	Cross-sectional	VA administrative data linked with WATCH and Area Resource Files	Women veterans with at least one VA primary care visit in FY17	*n* = 408,482 (American Indian or Alaska Native *n* = 5084, White *n* = 226,343, unknown *n* = 14,176, Native Hawaiian or other Pacific Islander *n* = 4156, Hispanic/Latinx *n* = 26,442, Black *n* = 124,370, Asian *n* = 5911)	Living in a gynecological supply desert (Women were assigned a level of community gynecologist supply corresponding to their county of residence per VA Enrollment file data)	Subpopulations with the highest proportions of women who lived in a gynecologist supply desert included those with American Indian or Alaska Native race
Process	Ryan (2016)^79^	Cross-sectional	Survey data	Women veterans ≤52 years old enrolled at one of 2 Midwestern VA medical centers in the 5 years prior to, or during July 2005–August 2008	*n* = 989 (non-Hispanic/Latinx White *n* = 790, non-White Race/Ethnicity (including Hispanic/Latinx) *n* = 199. Race and ethnicity were combined.	Ever had a hysterectomy	No difference in history of hysterectomy by race/ethnicity: 20% of veterans with no hysterectomy were non-White and 21% of veterans with hysterectomy were non-White (*p* = 0.816)
Process	Callegari (2019)^80^	Cross-sectional	VA administrative data	Veterans with a hysterectomy provided or paid for by VA 2012–2014	*n* = 2548 (non-Hispanic/Latinx White *n* = 1317, non-Hispanic/Latinx Black *n* = 1089, Hispanic/Latinx *n* = 142)	Hysterectomy route (abdominal, laparoscopic with or without robotic assist, vaginal)	Among those with fibroids, non-Hispanic/Latinx Black veterans were less likely than non-Hispanic/Latinx White veterans to have a laparoscopic versus abdominal hysterectomy (RRR 0.57; 95% CI: 0.44–0.77) or a vaginal versus abdominal hysterectomy (RRR 0.66; 95% CI: 0.49–0.88). No racial/ethnic differences detected among those without fibroids
Process	Bossick (2020)^81^	Cross-sectional	VA administrative data	Black and White veterans with a hysterectomy for benign indications provided or paid for by VA 2007–2014	*n* = 6785 (≤50 year old *n* = 5565 [Black *n* = 2527, White *n* = 3039]; >50 years old *n* = 1220 [Black *n* = 379, White *n* = 841])	Hysterectomy with concomitant bilateral salpingo-oophorectomy (BSO)	Among those ≤50 years old, in comparison to White veterans, Black veterans had lower odds of BSO (adjusted odds ratio [aOR]: 0.60; 95% CI: 0.51–0.69). No association between race and BSO was observed among those >50 years old.
Process	Carey (2023)^82^	Qualitative	Semi-structured interviews	Self-identified Black veterans ages 18–54 with newly diagnosed symptomatic uterine fibroids between FY10-FY13	*n* = 20	Black veterans’ experiences of care for uterine fibroids from symptom onset through diagnosis and treatment, including the role of experiences of racism and bias. No participants identified as Hispanic/Latinx.	Emergent themes identified: (1) The impact of severe fibroid symptoms in male-dominated military culture and beyond; (2) multilevel barriers to receiving timely, high-quality care for fibroids; (3) insufficient treatment options and need for self-advocacy to obtain desired treatments; (4) interpersonal racism and provider bias; and (5) impact of fertility loss owing to fibroids on mental health and relationships with partners
Process	Katon (2023)^11^	Cohort/longitudinal	VA administrative data	Black and White 18- to 54-year-old veterans using VA with newly diagnosed symptomatic uterine fibroids between fiscal year (FY) 2010 and FY12	*n* = 8247 (<45 years old: Black *n* = 3115, White *n* = 1649; ≥ 45 years old: Black *n* = 1926, White *n* = 1557)	1) Receipt of fibroid treatment (any versus none), 2) hysterectomy as first fibroid treatment (yes/no)	After adjustment among those with anemia who were <45 years old, 60.3% of Black veterans (95% CI: 56.9–63.6) versus 70.5% of White veterans (95% CI: 65.8–75.3) received any treatment for uterine fibroids. Among those without anemia who were <45 years old, 51.6% of Black veterans (95% CI: 48.2–55.1) versus 56.0% of White veterans (95% CI: 53.1–59) received any treatment for uterine fibroids. Regardless of age or anemia status Black veterans were less likely than White veterans to have a hysterectomy as their first treatment

#### Gynecology | structural (n = 2) and process (n = 5) measures

Structural measures included residing in a “gynecology desert” where the closest VA did not offer gynecological care and supply in the community was minimal^77^ and having a hysterectomy at a VA site with organizational characteristics thought to promote access to minimally invasive hysterectomy.^78^ In terms of gynecology process outcomes, all of the articles focused on hysterectomy or uterine fibroids,^79–83^ with all but one article^79^ coming from a single research group. This was the only topic area that included a qualitative study. The single qualitative article focused on the experiences of Black veterans with uterine fibroids, highlighting challenges in receiving timely and appropriate uterine fibroid care, particularly if they wished to avoid hysterectomy.^82^ These qualitative findings potentially provide additional context and explanation for results from the quantitative articles.

### Reproductive health screenings, menopause, and infertility

Table 5 includes findings from other topic areas with fewer than five articles each, including reproductive health screenings (*n* = 3),^84–86^ menopause (*n* = 3),^87–89^ and infertility (*n* = 3).^35,90,91^

**Table 5.** Articles that Addressed Measures of Health Care Quality for Reproductive Health Screenings, Menopause, and Infertility, by Topic, Measure Type, and Year

Topic, measure type	Primary author (year)	Study design	Data source	Population	*N*	Outcome(s)	Findings
Reproductive health screenings, Process	Reddy (2019)^85^	Cross-sectional	VA administrative data	Women veterans receiving veterans Affairs care between 2001 and 2014 derived from the Women veteran’s Cohort Study.	*n* = 113,796 (non-Hispanic/Latinx White *n* = 57,123, non-Hispanic/Latinx Black *n* = 32,104, Hispanic/Latinx *n* = 13,192, other *n* = 8823, missing *n* = 2554)	Receipt of HIV screening	Compared with non-Hispanic/Latinx White veterans, non-Hispanic/Latinx Black (aOR 1.6; 95% CI: 1.6–1.7), Hispanic/Latinx (aOR 1.4; 95% CI: 1.3–1.4), and other (aOR 1.4; 95% CI: 1.3–1.5) veterans were more likely to receive HIV screening
Reproductive health screenings, Process	Kedem (2022)^86^	Cohort/longitudinal	VA administrative data	Women veterans in VA care 2019 <25 years old	*n* = 16,102 (White *n* = 8306, Black *n* = 4248, Asian *n* = 293, Native American *n* = 183, Native Hawaiian or Pacific Islander *n* = 234, unknown *n* = 2367, Multiracial *n* = 471; Hispanic/Latinx ethnicity *n* = 2613)	Screening for gonorrhea or chlamydia	Compared with White veterans, Black veterans were more likely to be screened for gonorrhea or chlamydia (aOR 2.11; 95% CI: 1.89–2.36); compared with those who were not Hispanic those who were Hispanic were more likely to be screened for gonorrhea or chlamydia (aOR 1.30; 95% CI: 1.15–1.49)
Reproductive health screenings, Process	Ferras (2023)^84^	Cross-sectional	Survey data	Convenience sample of women veterans ≥18 years old separated from the military for at least a year	*n* = 90 (non-Hispanic White *n* = 60, other *n* = 30)	Being up to date on cervical cancer screening and mammography based on age recommendation	No association between race/ethnicity and screening OR 0.49 (0.14–1.77)
Menopause, Process	Haskell (2008)^88^	Cohort/longitudinal	VA Administrative Data	Women veteran VA users with prescriptions for hormone therapy in 2001 (either estrogen/progesterone combination or estrogen alone)	*n* = 36,222 (non-Hispanic/Latinx White *n* = 21,086, non-Hispanic/Latinx Black *n* = 4485, Hispanic/Latinx *n* = 723, other/unknown *n* = 9928	Discontinuation of hormone therapy in 2003/2004 based on pharmacy data	Compared with non-Hispanic/Latinx White veterans, those of Hispanic ethnicity (aOR, 1.41; 95% CI: 1.19–1.67), non-Hispanic/Latinx Black race (aOR, 1.13; 95% CI: 1.05–1.22), or other/unknown race (aOR, 1.08; 95% CI: 1.02–1.14) were more likely to discontinue hormone therapy
Menopause, Process	Gerber (2015)^87^	Cross-sectional	VA Administrative Data	Women veterans ≥45 years old using VA care in FY09	*n* = 157,195 (White *n* = 95,878, Black *n* = 39,909, Hispanic/Latinx *n* = 9844, other *n* = 11,564)	Use of postmenopausal hormone therapy (receipt or listed use of any oral or transdermal estrogen-containing product, combined estrogen/progestin product not used for contraceptive purposes during FY09)	Compared with White veterans, Black (aOR 0.66; 95% CI: 0.64–0.69) and Hispanic/Latinx veterans (aOR 0.65; 95% CI: 0.61–0.70) were less likely to receive postmenopausal hormonal therapy
Menopause, Process	Blanken (2022)^89,a^	Cross-sectional	VA Administrative Data	Women veterans 45 to 64 years with ≥ VA outpatient encounter in FY 2014 and/or 2015.	*n* = 200,901 (non-Hispanic White *n* = 116,128, non-Hispanic Black *n* = 65,215, Hispanic/Latinx *n* = 8466, other *n* = 7420, missing *n* = 12,138)	Prescription for systemic menopausal hormone therapy (HT); prescription for vaginal estrogen HT)	Non-Hispanic/Latinx Black (OR 0.74; 95% CI: 0.70–0.77, *p* < 0.001) and Hispanic/Latinx (OR 0.68; 95% CI: 0.61–0.77, *p* < 0.001) women had lower odds of systemic HT compared with non-Hispanic/Latinx White women; non-Hispanic/Latinx Black women (OR 0.78; 95% CI: 0.74–0.81) and Hispanic/Latinx women (OR 1.12; 95% CI: 1.02–1.24, *p* < 0.05) had lower odds of vaginal estrogen HT compared with non-Hispanic/Latinx White women
Menopause, Clinical or behavioral	Blanken (2022)^89,a^	Cross-sectional	VA Administrative Data	Women veterans 45 to 64 years with ≥ VA outpatient encounter in FY 2014 and/or 2015.	*n* = 200,901 (non-Hispanic White *n* = 116,128, non-Hispanic Black *n* = 65,215, Hispanic/Latinx *n* = 8466, other *n* = 7420, missing *n* = 12,138)	Menopause symptoms (ICD-9s for menopause-related diagnoses at two or more VA encounters in 2014–2015); prescription for systemic menopausal hormone therapy (HT); prescription for vaginal estrogen HT)	Non-Hispanic/Latinx Black women veterans had lower odds of documented menopause symptoms relative to non-Hispanic/Latinx White women veterans (OR 0.82; 95% CI: 0.78–0.86)
Infertility, Process	Mattocks (2015)^91,a^	Cross-sectional	VA Administrative data	OEF/OIF/OND women veterans of childbearing age who used VA medical or mental health care at least once between October 1, 2001 and December 30, 2010	*n* = 68,442 (non-Hispanic/Latinx White *n* = 32,880, non-Hispanic/Latinx Black *n* = 17,234, Hispanic/Latinx *n* = 7333, other *n* = 5417)	Infertility evaluation by CPT	No differences by race were detected for infertility evaluation (*p* = 0.21)
Infertility, Process	Goosen (2019)^90,a^	Cross-sectional	Survey data	OEF/OIF veterans who were enrolled for care in the New England Region or Indiana	*n* = 1004 (White *n* = 802, Racial Minority = 202)	Evaluation for infertility (yes, no), treatment for infertility (yes, no)	Compared with White veterans, racial minority veterans who were evaluated for infertility were less likely to report infertility treatment (aOR 0.24; 95% CI: 0.09–0.68). No differences were detected by race for infertility evaluation (aOR 0.98; 95% CI: 0.49–1.95)
Infertility, Clinical or behavioral	Mattocks (2015)^91,a^	Cross-sectional	VA administrative data	OEF/OIF/OND women veterans of childbearing age who used VA medical or mental health care at least once between October 1, 2001 and December 30, 2010	*n* = 68,442 (non-Hispanic/Latinx White *n* = 32,880, non-Hispanic/Latinx Black *n* = 17,234, Hispanic/Latinx *n* = 7333, other *n* = 5417)	Infertility diagnosis by ICD-9; infertility evaluation by CPT	Black veterans more likely than White veterans to have an infertility diagnosis (2.6% versus 1.5%, *p* < 0.001)
Infertility, Clinical or behavioral	Goosen (2019)^90,a^	Cross-sectional	Survey data	OEF/OIF veterans who were enrolled for care in the New England Region or Indiana	*n* = 1004 (White *n* = 802, Racial Minority = 202)	Infertility (“have you ever tried to have a baby but couldn’t?”), evaluation for infertility (yes, no), treatment for infertility (yes, no)	Racial minority veterans were more likely to have experienced infertility relative to White veterans (aOR 1.50; 95% CI: 1.02–2.22)
Infertility, Clinical or behavioral	Mancuso (2022)^35^	Cohort/longitudinal	Survey data	Female and male veterans 20–45 years old separated from service ≤10 years	Female veterans *n* = 1194 (non-Hispanic/Latinx White *n* = 644, non-Hispanic/Latinx Black *n* = 149, Hispanic/Latinx *n* = 61, other *n* = 340)	Infertility (no pregnancy after ≥12 months unprotected intercourse)	Women veterans with infertility were more likely to be non-Hispanic/Latinx Black versus non-Hispanic/Latinx White or Hispanic/Latinx (10.8% versus 14.2% *p* = 0.04)

^a^ Study included multiple types of measures.

#### Reproductive health screenings, menopause, and infertility | process measures (n = 9)

Data on reproductive health screenings, including being up to date on cervical cancer screening or mammography^84^ and frequency of screening for sexually transmitted infection (STI) screenings,^85,86^ were derived from electronic health records. Articles on menopause examined receipt of a prescription for hormone therapy (HT)^87,89^ and discontinuation of HT following publication of the initial findings from the Women’s Health Initiative study, which indicated that HT use was associated with increased risk for breast cancer and cardiovascular disease.^88,92^ Articles examining process measures related to infertility included receipt of infertility evaluation or treatment and highlighted challenges of comparing measures for infertility care derived from electronic health records versus survey data, as frequencies varied considerably depending on how these measures were operationalized.^90,91^

##### Reproductive health screenings, menopause, and infertility | behavioral or clinical measures (n = 4)

Articles in this category included one that measured diagnosed menopausal symptoms documented in the electronic health record^89^ and three articles addressing infertility, including two using self-reported diagnosis of infertility^35,90^ and one that relied on documentation in the electronic health record.^91^

## Discussion

Using Donabedian’s model for the measurement of health care,^30^ this scoping review examined racial and ethnic disparities in reproductive health care and outcomes among female veterans and found that, despite a growth in the literature, there were significant limitations in published data. A minority of studies included race or ethnicity as primary independent variables or explicitly mentioned racism. Fewer still provided any data or explanation for the mechanisms through which racism operated to generate the observed disparities. Additionally, almost no articles examined structural measures, and none included measures of systemic racism. Significant topical gaps in the literature were identified, with few articles that addressed reproductive health screening, menopause, or infertility and none that examined racial disparities related to breast or gynecological cancer, sexual function, or pelvic floor disorders, reflecting overall gaps in the literature with respect to these topics and female veterans.^93^ Finally, there was significant variation in study design and operationalization of outcome measures across all topics.

VA studies examining a variety of health care settings and specialties suggest that while the enhanced access to health care afforded to veterans in VA can reduce racial and ethnic disparities in process measures, racial and ethnic disparities in clinical outcomes are not necessarily reduced.^94–96^ Consistent with this literature, we found that across reproductive health topics, racial and ethnic disparities were less frequently detected for process measures versus clinical and behavioral outcome measures. While veterans using VA care have enhanced access due to minimum or no co-pays and travel assistance, access alone cannot undo the impact of a lifetime of exposure to structural and interpersonal racism or prevent harm due to biased or discriminatory treatment.^9,57,97–100^ Additionally, access is a multidimensional construct consisting of more than just financial facilitators or barriers and is distinct from utilization.^101^ Thus, to more deeply examine potential racial disparities in process measures, studies are needed that operationalize measurement of access across multiple dimensions, such as temporal (are appointments available at conventional times) or cultural (trust in the health care system or providers), which likely vary by race and ethnicity.^101^

Study findings also highlight the need to explore process measures in terms of patient preferences and experience, particularly to provide context and to identify culturally competent for racial and ethnic disparities in process and clinical and behavioral outcomes. For example, Black and Hispanic veterans, compared with White veterans, were less likely to have a documented contraceptive method and less likely to report using an IUD or implant at last intercourse.^60,69^ However, a separate article reported that Black and Hispanic veterans were more likely than White veterans to prefer nonhormonal methods and methods that also prevented STIs.^62^ Veterans of minoritized racial and ethnic groups were also more likely than White veterans to report a mismatch between their ideal and current contraceptive method and had lower contraception knowledge scores.^67^ Thus, inclusion of data on patient preferences and experiences suggests that access as measured by availability of a full range of contraceptive methods at every VA may be less of an issue than lack of access to culturally competent patient-centered care. Nevertheless, there was a significant gap in the literature regarding veterans’ preferences and experiences with VA reproductive health care, whether these varied by race/ethnicity, and how or if they impacted clinical and behavioral outcomes.

Strengths of this scoping review included a group of co-authors with expertise in reproductive health and women veterans’ health research, using an established set of search terms, and applying the Donabedian’s framework for measuring health care quality to inform the synthesis of results. However, several limitations are important to consider. Most articles did not include race or ethnicity as primary predictor variables; thus, models and study designs were not necessarily designed to specifically address issues of racial and ethnic disparities and health equity and may have had limited power to detect racial and ethnic disparities. Authors also frequently relied on categorizations such as “non-White” or “racial minority” rather than more specific groupings. Relatedly, the conceptualization of race or ethnicity and how it was incorporated into the analyses and discussion of results was frequently limited, with few studies explicitly addressing racism as a root cause or incorporating measures of structural, systemic, or interpersonal racism. Nearly all articles focused exclusively on veterans using VA care, limiting generalizability to the broader population of veterans. The majority of articles on contraception and pregnancy relied on data from two large VA studies. While these studies provide rich data sources for secondary analyses, they also have their own limitations, raising questions regarding the robustness of some findings, as was highlighted by conflicting results from studies using different methodologies or sampling frames. Additionally, no studies examined differences in racial and ethnic disparities in services provided by VA versus those purchased from the community. Finally, due to the heterogeneity in study measures and racial and ethnic group categorizations, the data were not well suited for quantitative synthesis or meta-analysis. Nevertheless, our findings in this review provide important information suggesting areas where VA may need to focus efforts to improve health equity in reproductive health care and where more research is needed.

### Implications for equity

Concurrent with the growth in the female veteran population and increasing availability of reproductive health care in VA, there is a steady growth in the literature regarding racial and ethnic disparities in this care and outcomes.^21,22,102^ While not amenable to meta-analysis due to heterogeneity of outcome measurement, there is sufficient literature to warrant systematic reviews for some topic areas (e.g., contraception and pregnancy). Notably, much of this literature results from secondary analyses from a relatively small number of datasets, many of which were not explicitly designed to address questions of health equity. Thus, there is a need for multidisciplinary studies of reproductive health among female veterans that are intentionally grounded in principles and frameworks of health equity and engage with veterans to understand the causes of racial and ethnic disparities in reproductive health care and outcomes among veterans.^103–107^ Research is also needed that measures multidimensional aspects of access as well as veterans’ preferences and experiences. Findings from such studies can provide deeper understanding of racial and ethnic disparities in reproductive health care and outcomes among veterans and inform the development and testing of interventions to reduce or eliminate disparities.

## Conclusions

Understanding the causes of racial and ethnic health disparities and their relationships with different measures of health care quality is essential for achieving health equity for female veterans. Despite the rapid growth in the literature regarding women veterans’ health, there are significant gaps in understanding racial and ethnic disparities in reproductive health and outcomes of women veterans and substantial heterogeneity in the literature in terms of study designs and how outcomes are defined and measured.

## Notes

^a^
Given that sex refers to biology, and this review deals with preventive care and disease and treatment for female reproductive organs we use the term “female veterans” throughout this article rather than “women veterans.” Where possible, we have also used gender neutral terms.

## Abbreviations Used

95% CI: 95% Confidence Interval
aOR: Adjusted odds ratio
AI/AN: American Indian/Alaska Native
BRFSS: Behavioral Risk Factor Surveillance System
CDC: Centers for Disease Control and Prevention
CHERP: Center for Health Equity Research & Promotion
CPT codes: Current Procedural Terminology codes
ECUUN: Examining Contraceptive Use and Unmet Need
EPDS: Edinburgh Postnatal Depression Scale
FY: Fiscal year
HT: Hormone therapy
ICD-9: International Classification of Disease, Ninth Revision
IUD: Intrauterine device
MISSION Act: Maintaining Internal Systems and Strengthening Integrated Outside Networks Act
MPR: Medication possession ratio
OR: Odds ration
OEF: Operation Enduring Freedom
OIF: Operation Iraqi Freedom
OND: Operation New Dawn
PRISMA: Preferred Reporting Items for Systematic Reviews and Meta-Analyses
PPA: Postpartum anxiety
PPD: Postpartum depression
PPPTSD: Postpartum posttraumatic stress disorder
PTSD: Posttraumatic Stress Disorder
VA: Veterans Health Administration
WATCH: Women’s Assessment Tool for Comprehensive Health
WVCS: Women Veterans Cohort Study
