# Supplementary material for: Racial and Ethnic Disparities in Reproductive Health Care and Outcomes Among Female Veterans: A Scoping Review
Source: Health Equity. 2025 Apr 10;9(1):203–28. doi: 10.1089/heq.2024.0168 (PMC12270534; doi:10.1089/heq.2024.0168)
Supplement: Supplementary Data [file heq.2024.0168_supplementary_data.docx]

| **Manuscript Information** | |
| --- | --- |
| **Title:** |  |
| **1^st^ Author:** |  |
| **PMID** |  |
| **Include:** | [ ] Yes  [ ] No (If exclude please indicate why below, chose the most relevant option) |
| **Exclude:** | [ ] Not original research (e.g. editorial, review, expert opinion)  [ ] Not related to reproductive health/reproductive health services (Reproductive health, Pregnancy, Infertility, Sexually transmitted infections, Menopause, Menopause symptoms, Hormone treatment, Gynecology, Contraception, reproductive health care use)  [ ] Not women Veterans OR insufficient inclusion of women Veterans  [ ] Veterans from another country  [ ] Active duty women only  [ ] Included too small a proportion or absolute number of women Veterans (e.g. total N<100 and proportion of women <10%, total N = 100-1000 and proportion of women <5e)  [ ] Total proportion Veterans <75% and does not explicitly address results for Veterans  [ ] Total proportion women <75% and does not explicitly address results for women  [ ] Does not include reporting of outcome(s) by race/ethnicity OR does not report measures of association of race/ethnicity and outcome(s) (e.g. odds ratios, hazard ratios, relative risks, etc) |
|  | **NOTE:** We will include studies that only classify Veterans as white or non-White |

| **Study Information** | |
| --- | --- |
| **Study Design:**  **NOTE no RCTs were identified for review** | [ ] RCT or implementation trial  [ ] Cross-sectional/Descriptive (observational)  [ ] Cohort/Longitudinal (observational)  [ ] Case-Control (observational)  [ ] Other (e.g. qualitative, implementation study)  Describe: |
| **Research Question/Objective:** |  |
| **Study Population:** (describe the target population, e.g. VA users, rural Veterans, etc.) |  |
| **Inclusion/Exclusion Criteria:** (list specific criteria) |  |
| **Total N in analytic sample:** |  |
| **Racial and/or ethnic categories used and N per category in study sample (if ethnicity was separate from race include both breakdowns, but indicate this):** |  |

| **Study Measures** | |
| --- | --- |
| **Was Race or ethnicity the primary independent/predictor/exposure variable?** | [ ] Yes  [ ] No  Comment: |
| **How was race and/or ethnicity determined (e.g. self-report, medical records, clinician report)?** |  |
| **Was there any language in the intro, methods, or discussion about how race or ethnicity were defined or conceptualized?** | [ ] Yes  [ ] No  Comment: |
| **Outcome (s):** (include information for each outcome) |  |
| **Description** (describe outcome variable(s)) |  |
| **Measurement** (describe how measurement of the outcome(s) was operationalized. Relevant information could include the scale that was used, timing of the measurement, methods of data collection, any validity information, etc) |  |
| **N with outcome(s):** |  |
| **Was prevalence of outcome(s) listed by race/ethnicity:** | [ ] Yes  [ ] No  Comment: |
| **Additional Comments:** |  |

| Analysis | |
| --- | --- |
| **Statistical methods:** (describe what statistical methods were used, including statistical tests, regression methods, or others) |  |
| **Additional Comments:** |  |

| Results and Conclusion | |
| --- | --- |
| **Main results regarding frequency of outcome by race/ethnicity or measures of association:** (provide primary results, this may include percentages, p-values, relative risks, odds ratios, etc, whenever possible include 95% confidence intervals, ensure that the reference group or groups being compared is specified) |  |
| **Conclusion:** |  |
| **Additional Comments:** |  |

| Limitations | |
| --- | --- |
| **Key limitations, including those identified by the reviewer but potentially not listed by the authors:** |  |
